# The impact of using self-report versus objective measures of cardiometabolic conditions in epidemiologic research: a case study from India using data from the longitudinal aging study in India

**DOI:** 10.3389/fepid.2024.1372972

**Published:** 2025-07-25

**Authors:** Emma Nichols, Peifeng Hu, David E. Bloom, Jinkook Lee, T. V. Sekher

**Affiliations:** ^1^ Center for Economic and Social Research, University of Southern California, Los Angeles, CA, United States; ^2^ Division of Geriatric Medicine, University of California, Los Angeles, CA, United States; ^3^ Department of Global Health and Population, Harvard School of Public Health, Boston, MA, United States; ^4^ Department of Economics, University of Southern California, Los Angeles, CA, United States; ^5^ Department of Family and Generations, International Institute for Population Sciences, Mumbai, India

**Keywords:** high blood pressure, diabetes, measurement, global health, bias, epidemiology

## Abstract

**Introduction:**

In low- and middle-income countries, self-reported data on chronic cardiometabolic conditions such as high blood pressure and diabetes are commonly used in large-scale epidemiologic studies because implementing objective measures is challenging in these contexts. However, existing evidence suggests that the sensitivity of such measures may be low, and performance may differ by factors such as age, education, or income. We sought to confirm these prior findings and assess bias due to the use of self-reported data in hypothetical epidemiologic studies considering high blood pressure and diabetes as exposures, outcomes, and confounders.

**Methods:**

We used data from the Longitudinal Aging Study in India (analytic *N* = 55,392) to assess the performance of self-reported data on high blood pressure and diabetes compared with objective measures, overall and stratified by basic demographic factors. We then compared regression coefficients from models considering self-reported and objective high blood pressure and diabetes as exposures, outcomes, and confounders. In all models, we examined whether the mode of data collection (self-report or objective) for other key variables in the model affected results.

**Results:**

The overall sensitivity of self-reported high blood pressure and diabetes was 0.514 and 0.570, respectively; specificity for the two conditions was 0.922 and 0.984. Sensitivity of both conditions increased with age, and was higher among women, those in urban settings, and those with higher educational attainment. Across almost all models considering high blood pressure and diabetes as either exposures or outcomes anti-conservative bias was observed when using self-reported vs. objective measures, regardless of the mode of data collection for other key variables. When high blood pressure and diabetes were considered as confounders, differences between using self-report and objective measures were minimal.

**Discussion:**

Anti-conservative bias due to the use of self-reported measures of chronic cardiometabolic conditions in surveys conducted in low- and middle-income contexts may be common. Future studies may seek to quantify the magnitude of anticipated bias in existing data resources and use quantitative bias analysis to formally estimate the potential implications of misclassification.

## 1 Introduction

Chronic conditions such as high blood pressure and diabetes mellitus are highly prevalent and are leading causes of health loss globally (1–3). Dual demographic and epidemiologic transitions have led to increases in the relative importance of these conditions in low- and middle-income contexts (4, 5). However, the objective assessment of blood pressure and diabetes can be challenging in such contexts due to high costs and logistical barriers. These challenges are further amplified in large, nationally representative surveys, which are a crucial resource for conducting generalizable epidemiologic research.

Instead, self-report measures are more commonly used in large-scale surveys administered in low- and middle-income settings. However, many studies have previously shown substantial discordance between self-report and objective measures of high blood pressure and diabetes. Reported sensitivities for objective high blood pressure based on self-report data range from 50% to 80% across settings (6–11). In studies examining both high blood pressure and diabetes, sensitivity for objective diabetes based on self-report was somewhat higher (61%–85%) than for high blood pressure (50%–73%), although substantial misclassification remained (8–10). In most studies, reported specificity for both conditions was high (>95%), although one study reported 90% specificity for blood pressure (11). Although 90% specificity is relatively high, specificities in this range can still lead to substantial misclassification when a given outcome is rare.

Prior work has also shown that the sensitivities of self-report measures of blood pressure and diabetes vary by demographic factors and indicators of socioeconomic status (SES) (8, 9, 12–17). Research across settings including Indonesia, India, China, England, and Ireland has found that those with lower educational attainment or income are less likely to self-report having high blood pressure or diabetes given that they have tested positive on objective measures (i.e., self-report has lower sensitivity in these groups) (8, 9, 12, 14, 16, 17). The majority of studies suggest that self-report measures have lower sensitivity for younger participants (6, 8, 10, 15), although some evidence indicates that sensitivity of self-report measures may also be worse for those in the oldest age categories (10, 13).

Given these observed differences, considering the potential impact of measurement error is important when analyzing data on self-reported blood pressure or diabetes in epidemiologic studies. Because most exposures considered in epidemiologic studies are correlated with the same demographic and SES-related factors associated with measurement error in self-reported blood pressure and diabetes, measurement error is likely to differ by exposure status and can therefore lead to bias in any direction when considering blood pressure or diabetes as outcomes of interest (18). Prior studies in the United States have found that using self-report instead of objective measures of physical activity or hearing impairment can lead to meaningful bias in epidemiologic research (19, 20). One study in Canada examined the impact of using self-reported high blood pressure as an outcome and found that because the sensitivity of high blood pressure was higher in obese individuals, the association between obesity and high blood pressure was overestimated when using self-reported high blood pressure instead of objectively measured high blood pressure (21).

However, to our knowledge, no existing study has estimated the potential biases associated with using self-reported high blood pressure and diabetes status in epidemiologic research in low- or middle-income contexts, where under-reporting is likely to be higher and may have a stronger gradient with SES-related factors (22). Considering how differential measurement error may further affect findings if self-report measures (such as blood pressure and diabetes) are used as exposures or hypothesized confounders in addition to outcomes in epidemiologic research is also important. In this work, we use data from the Longitudinal Aging Study in India (LASI) to directly compare self-reported and objective measures of chronic conditions and understand the effect of using self-reported data on high blood pressure and diabetes in epidemiologic studies in low- and middle-income contexts. We explore the impact of using self-report measures when considering blood pressure and diabetes as exposures, outcomes, and confounders and consider how differences in the mode of data collection (self-report vs. objective measures) for other key variables in the model may affect results.

## 2 Methods

### 2.1 Sample

LASI includes a nationally representative sample of adults aged 45 years and older and their spouses in India. The survey was administered to more than 73,000 adults in 2017–2019, and collected data included demographics, self-reported health conditions, physical measurements, biomarkers, and measures of cognitive functioning. Informed consent was obtained from all participants (written or thumb impression), and the study was approved by the relevant institutional review boards. This analysis excluded all spouses of sampled respondents who were under 45 (*N* = 6,789) and those without dried blood spot samples (*N* = 8,233), which were required for the objective assessment of diabetes. We also excluded those with further missing data on either objective diabetes measurement (*N* = 42), objective blood pressure measurement (*N* = 46), self-reported high blood pressure (*N* = 2), or covariates included in regression analyses (*N* = 2,904), as data on these variables was needed in primary analyses (details in Supplementary Material). The analytic sample size was *N* = 55,392 (total N excluded = 18,016). Of those aged 45 and older, those who were excluded were slightly older (mean age: 62.0 for excluded vs. 59.3 years for included), less likely to live in rural areas (% rural: 58% for excluded vs. 66% for included) and had higher educational attainment (% in graduate school: 7% for excluded vs. 5% for included) (unweighted comparisons). There were no differences in the gender of participants who were included vs. excluded.

### 2.2 Objective measures of high blood pressure and diabetes

Systolic and diastolic blood pressure were assessed objectively using an automatic device (Omron HEM 7121). Three readings of systolic and diastolic blood pressure were taken with one-minute intervals between consecutive measures; the average of the second and third readings was used to estimate blood pressure. Objective high blood pressure was defined as systolic blood pressure greater than or equal to 140 mmHg, diastolic blood pressure greater than or equal to 90 mmHg, or self-reported use of blood pressure-lowering medications, in line with clinical practice guidelines in India (23). In a sensitivity analysis, we compared findings from primary analyses to results using the new blood pressure guidelines from the American Heart Association (AHA) (systolic blood pressure greater than or equal to 130 mmHg and diastolic blood pressure greater than or equal to 80 mmHg) (24).

We used dried blood spot samples to measure hemoglobin A1c (HbA1c), a measure of average blood sugar levels over the past two to three months. HbA1c levels were assessed using the Cobas Integra 400 Plus Biochemistry analyzer (Roche Diagnostics, Switzerland) (25). We used a calibration process to convert HbA1c values based on dried blood spots to their whole blood equivalents. Of the participants, 87.7% consented to dried blood spot collection. We used a cutoff of 6.5% or higher to define diabetes (26). We also classified those who self-reported use of diabetes medications (injection or oral) as having objective diabetes.

Although including those who are on treatment in objective classifications is important because treatment can affect observed biomarkers, in LASI this partially conflates the objective and self-report measures because participants are only asked about treatment if they report diagnoses of high blood pressure or diabetes. Therefore, we also conducted sensitivity analyses using versions of the objective measures that do not consider self-reported medication use.

### 2.3 Self-report measures of high blood pressure and diabetes

Self-reports of high blood pressure and diabetes are based on questions asking respondents whether they had ever been told by a doctor that they had high blood pressure or diabetes.

### 2.4 Other important variables and covariates

Demographic variables and SES indicators considered throughout the analyses include self-reported age, gender, marital status (currently married or partnered/other), literacy (reported ability to read or write), caste (no caste or other caste/scheduled caste/scheduled tribe/other backwards class), and education (no school/less than secondary school/secondary or higher secondary school/graduate school). We also considered several health behaviors and risk factors, including self-reported smoking status (ever/never smoked or used smokeless tobacco), self-reported moderate and vigorous physical activity (every day/more than once per week/once per week/1–3 times per month/hardly ever or never), and body-mass index (BMI) category based on objectively measured height and weight (underweight: BMI <18.0 kg/m^2^; normal weight: BMI = 18.0–22.9 kg/m^2^; overweight: BMI = 23.0–24.9 kg/m^2^; obesity: BMI ≥25.0 kg/m^2^) (27). Finally, we used data on health outcomes, including self-report of a doctor-diagnosed heart problem (heart attack, congestive heart failure, or other chronic heart problems), self-reported difficulty in at least one activity of daily living (ADL) (walking across room, dressing, bathing, eating, getting in and out of bed, using the toilet) (28), difficulty balancing, and slow walking speed as important health outcomes. Difficulty balancing was defined as difficulty with any one of the following balance tests: side-by-side, semi-tandem, or full tandem. All balance tests required participants to hold each position for 10 s. Most individuals with difficulty balancing had difficulty with only the full tandem test (80%). Walking speed was measured using two timed trials across a 4-m uncarpeted area. Slow walking speed was defined as having an average speed across the two trials in the slowest 25% (≤0.7 m/s) of observed speeds.

### 2.5 Statistical analysis

We characterized the population using basic demographic variables and health-related risk factors and outcomes included in analyses using means and interquartile ranges for continuous variables and proportions for categorical and binary indicators. We then assessed the sensitivity, specificity, positive predictive value (PPV), and negative predictive value (NPV) of self-reported high blood pressure and diabetes compared with objective measures of blood pressure and diabetes, which were treated as the gold standard. We also estimated the prevalence of high blood pressure and diabetes using both self-report and objective measures.

We conducted three sets of analyses to assess the impact of using self-reported high blood pressure and diabetes instead of objective high blood pressure and diabetes in epidemiologic analyses. Set 1 considered blood pressure and diabetes as the exposures of interest, set 2 considered blood pressure and diabetes as the outcomes of interest, and set 3 considered blood pressure and diabetes as hypothesized confounders. Other variables were selected based on prior evidence of associations between example exposures and outcomes and to ensure that the temporal ordering of example analyses was appropriate. For example, in analyses where cardiometabolic conditions were considered as outcomes (set 2), we selected example exposures that would plausibly precede these conditions (i.e., self-reported physical activity instead of self-reported heart problem).

For all analyses, we considered whether impacts varied depending on whether other key variables included in the analysis were self-reported or objective measures. For set 1 of the analyses, we considered self-reported and objective outcomes (any ADL difficulty, difficulty balancing), for set 2 we considered self-reported and objective exposures (vigorous physical activity, overweight or obese BMI), and for set 3 we considered self-reported and objective exposures (self-reported heart problem, difficulty balancing) and outcomes (any ADL difficulty, slow walking speed). Models adjusted for demographics controlled for age (estimated with a natural cubic spline with two internal knots), gender, and educational attainment. Fully adjusted models across all analysis sets considered these additional confounders: state, rural/urban residence, marital status, literacy, self-reported smoking, and caste. Set 1 of the analyses additionally controlled for moderate and vigorous physical activity and BMI category, and set 3 additionally controlled for BMI category. All exposures and outcomes considered were binary. Therefore, across all analyses we used Poisson regression models with robust variance to estimate prevalence ratios.

All analyses and descriptive statistics used survey weights to account for unequal sampling probabilities. We used R version 4.2.2 for all analyses.

## 3 Results

### 3.1 Sample characteristics

We included 55,392 participants. About half (46.4%) of the weighted sample were women, and the mean age was 58.7 years (interquartile range: 50.0–65.0) (Table 1). Of the sample, 68.1% lived in rural areas and the majority (52.2%) had no formal education, while 21.4% had some formal education but less than secondary school, 21.3% had secondary and upper secondary school education, and 5.1% attended some graduate school. Of the weighted sample 13.3% had at least one ADL impairment, and 16.8% had difficulty on the balancing task.

**Table 1:** Demographics and health characteristics of the longitudinal aging study in India (LASI) (*N* = 55,392).

	LASI
N	55,392
Age	58.7 (50.0–65.0)
Women	46.4 (29,622)
Rural	68.1 (36,701)
Education	
No school	52.2 (26,007)
Less than secondary school	21.4 (13,831)
Secondary and higher secondary	21.3 (12,887)
Graduate school	5.1 (2,667)
High blood pressure	
Self-report	25.7 (15,667)
Objective	41.0 (24,546)
Diabetes	
Self-report	11.7 (6,894)
Objective	18.2 (10,521)
Any ADL impairment	13.3 (7,104)
Vigorous activity	39.1 (19,757)
Overweight/obese	41.7 (24,611)
Self-reported heart problem	3.5 (1,966)
Balance problem	16.8 (9,642)

Mean and IQR are shown for continuous variables, percentage and N are shown for binary or categorical variables. All statistics use survey weights to account for the complex survey design.

### 3.2 Prevalence of self-report and objective measures overall and by demographic characteristics

The prevalence of objective high blood pressure was 41.0% [95% Confidence Interval (CI) 40.5–41.6], whereas the prevalence of self-reported high blood pressure was substantially lower (25.7%; 95% CI 25.3–26.2) (Table 2). For both self-reported and objective high blood pressure, prevalence increased with age, was higher for women compared to men, those in urban compared to rural settings, and those with higher education. For diabetes, the prevalence of the objective measure (18.2%; 95% CI 25.3–26.2) was also higher than the self-reported measure (11.7%; 95% CI 11.4–12.0), although the difference was smaller. Again, patterns across demographic characteristics were consistent when comparing the objective measure to the self-report measure.

**Table 2:** The prevalence and performance of self-reported high blood pressure and diabetes compared to objective measures of disease in the longitudinal aging study in India (LASI) (*N* = 55,392).

	Self-report prevalence	Objective prevalence	Sensitivity	Specificity	Positive predictive value	Negative predictive value
Blood pressure						
Overall	25.7 (25.3–26.2)	41.0 (40.5–41.6)	0.514	0.922	0.820	0.731
Age category						
<45	17.4 (15.5–19.4)	28.6 (26.3–31.1)	0.417	0.924	0.688	0.798
45–59	21.7 (21.1–22.3)	36.1 (35.4–36.8)	0.468	0.925	0.779	0.755
60–74	32.3 (31.5–33.1)	49.4 (48.5–50.2)	0.569	0.916	0.868	0.685
75+	35.1 (33.3–37.0)	53.4 (51.4–55.3)	0.580	0.911	0.882	0.654
Gender						
Men	21.1 (20.4–21.7)	38.7 (37.9–39.5)	0.450	0.940	0.827	0.730
Women	31.1 (30.4–31.7)	43.7 (43.0–44.4)	0.579	0.898	0.816	0.733
Rurality						
Urban	34.1 (33.2–34.9)	50.5 (49.5–51.4)	0.599	0.923	0.888	0.693
Rural	21.8 (21.3–22.3)	36.6 (36.0–37.2)	0.459	0.921	0.771	0.747
Education level						
No school	23.4 (22.8–24.1)	38.2 (37.4–38.9)	0.480	0.917	0.782	0.741
Less than secondary school	26.9 (25.9–27.8)	42.8 (41.7–43.8)	0.528	0.925	0.840	0.724
Secondary and higher secondary	28.3 (27.3–29.4)	44.5 (43.4–45.7)	0.551	0.931	0.866	0.721
Graduate school	33.0 (30.8–35.4)	48.6 (46.1–51.0)	0.594	0.918	0.873	0.705
Diabetes						
Overall	11.7 (11.4–12.0)	18.2 (17.8–18.6)	0.570	0.984	0.890	0.911
Age category						
<45	6.5 (5.3–7.9)	14.1 (12.4–16.1)	0.418	0.993	0.910	0.912
45–59	10.3 (9.8–10.7)	16.9 (16.4–17.4)	0.541	0.986	0.890	0.914
60–74	14.9 (14.3–15.5)	21.1 (20.4–21.8)	0.627	0.979	0.886	0.907
75+	12.2 (11.0–13.5)	19.2 (17.7–20.8)	0.577	0.986	0.906	0.908
Gender						
Men	11.6 (11.1–12.1)	18.0 (17.4–18.6)	0.568	0.984	0.884	0.912
Women	11.8 (11.4–12.3)	18.5 (18.0–19.1)	0.573	0.985	0.898	0.910
Rurality						
Urban	19.3 (18.6–20.1)	28.2 (27.3–29.0)	0.637	0.981	0.928	0.873
Rural	8.1 (7.8–8.4)	13.6 (13.1–14.0)	0.505	0.986	0.848	0.927
Education level						
No school	8.0 (7.6–8.4)	13.6 (13.1–14.1)	0.495	0.986	0.846	0.925
Less than secondary school	13.7 (13.0–14.5)	20.7 (19.8–21.6)	0.598	0.983	0.901	0.904
Secondary and higher secondary	16.6 (15.8–17.5)	24.5 (23.6–25.5)	0.626	0.983	0.922	0.890
Graduate school	20.4 (18.5–22.4)	29.1 (26.9–31.3)	0.651	0.980	0.929	0.873

95% Confidence intervals are presented for estimates of prevalence. All reported statistics incorporate survey weights to account for the complex survey design.

### 3.3 Performance of self-report measures overall and by demographic characteristics

The overall sensitivity of self-reported high blood pressure and diabetes was 0.514 and 0.570, respectively (Table 2). For blood pressure, sensitivity increased with age, to 0.580 in older adults 75 years and older. Sensitivity of self-reported diabetes also increased with age but peaked in the 60–74 age group (0.627) and was lower for those 75 years and older (0.577). For both high blood pressure and diabetes, sensitivity was higher among women, those in urban settings, and those with higher levels of education.

Specificity was lower for high blood pressure (0.922) than for diabetes (0.984) (Table 2). For both conditions, differences across demographic groups were smaller. There was an 82.0% chance that someone who self-reported having high blood pressure had objectively measured high blood pressure (PPV) and a 73.1% chance that someone who self-reported not having high blood pressure did not have objectively measured high blood pressure (NPV). PPV and NPV were both higher for diabetes, although the difference was larger for NPV.

### 3.4 Impact when self-report measures were considered as exposures

**Figure 1:**
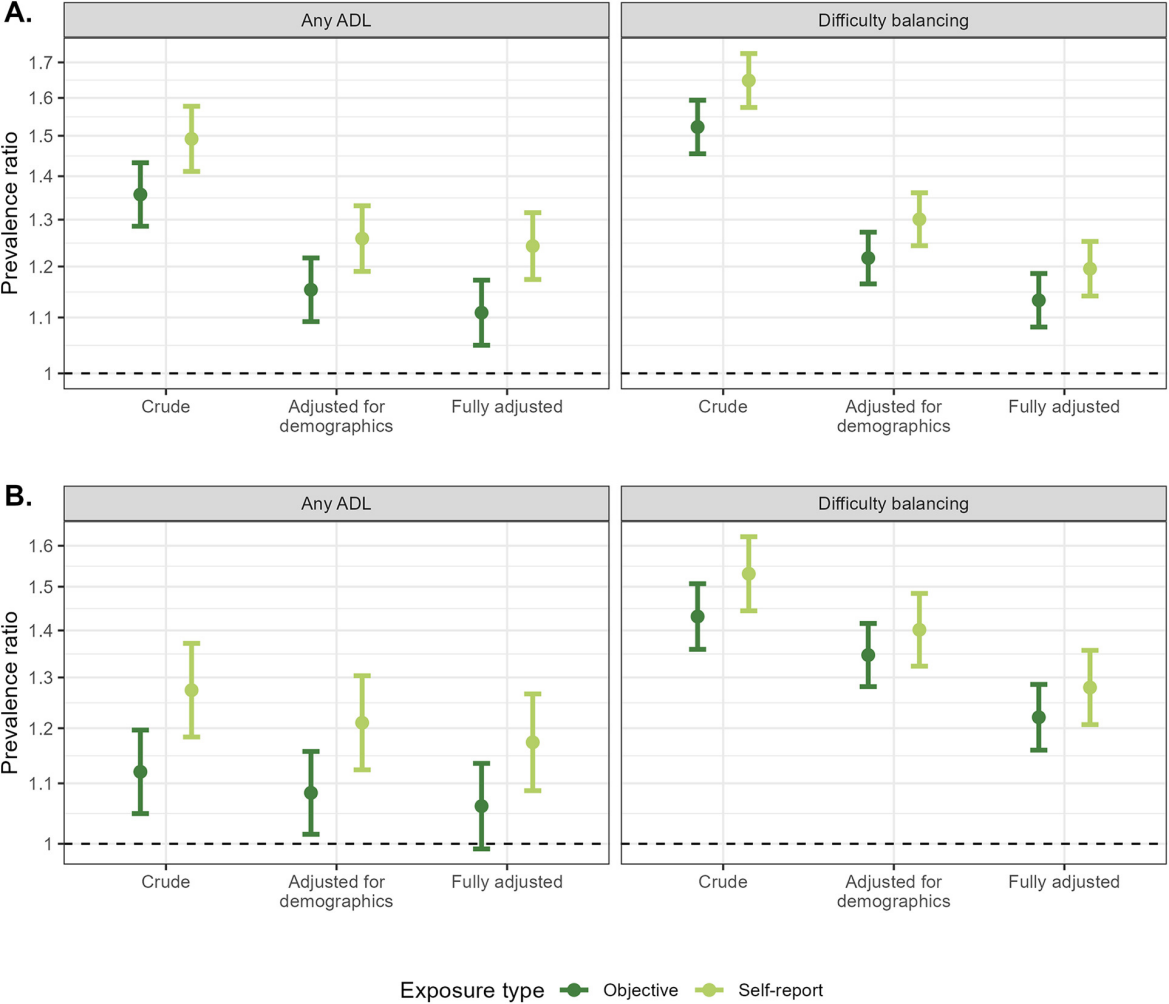
Comparisons of self-reported and objective high blood pressure **(A)** and diabetes **(B)** in models assessing the association between high blood pressure and diabetes with both a self-report [any activity of daily living (ADL) difficulty] and objective (difficulty balancing) health outcome. Models adjusted for demographics controlled for age (spline), gender, and educational attainment. Fully adjusted models additionally controlled for state, rural/urban residence, marital status, literacy, self-reported smoking, caste, moderate and vigorous physical activity, and BMI category.

Estimates of the associations between self-reported high blood pressure or diabetes and both self-report (any ADL) or objective (difficulty balancing) health outcomes were stronger than the same estimates using objective blood pressure or diabetes measures (Figure 1). Adjusting for potential confounders did attenuate all estimates but did not attenuate the observed difference between associations using self-report vs. objective measures of high blood pressure and diabetes. For fully adjusted models for blood pressure as an exposure, estimated associations were 109.89% and 43.22% higher using self-reported compared to objective blood pressure for any ADL impairment and difficulty balancing, respectively (Supplementary Table S1). For associations between diabetes and the same two health outcomes, associations were 169.74% and 23.54% higher for self-reported compared to objective measures. When assessing the association between diabetes and any ADL impairment, the association was statistically significant using the self-reported measure but not statistically significant when using the objective measure.

### 3.5 Impact when self-report measures were considered as outcomes

**Figure 2:**
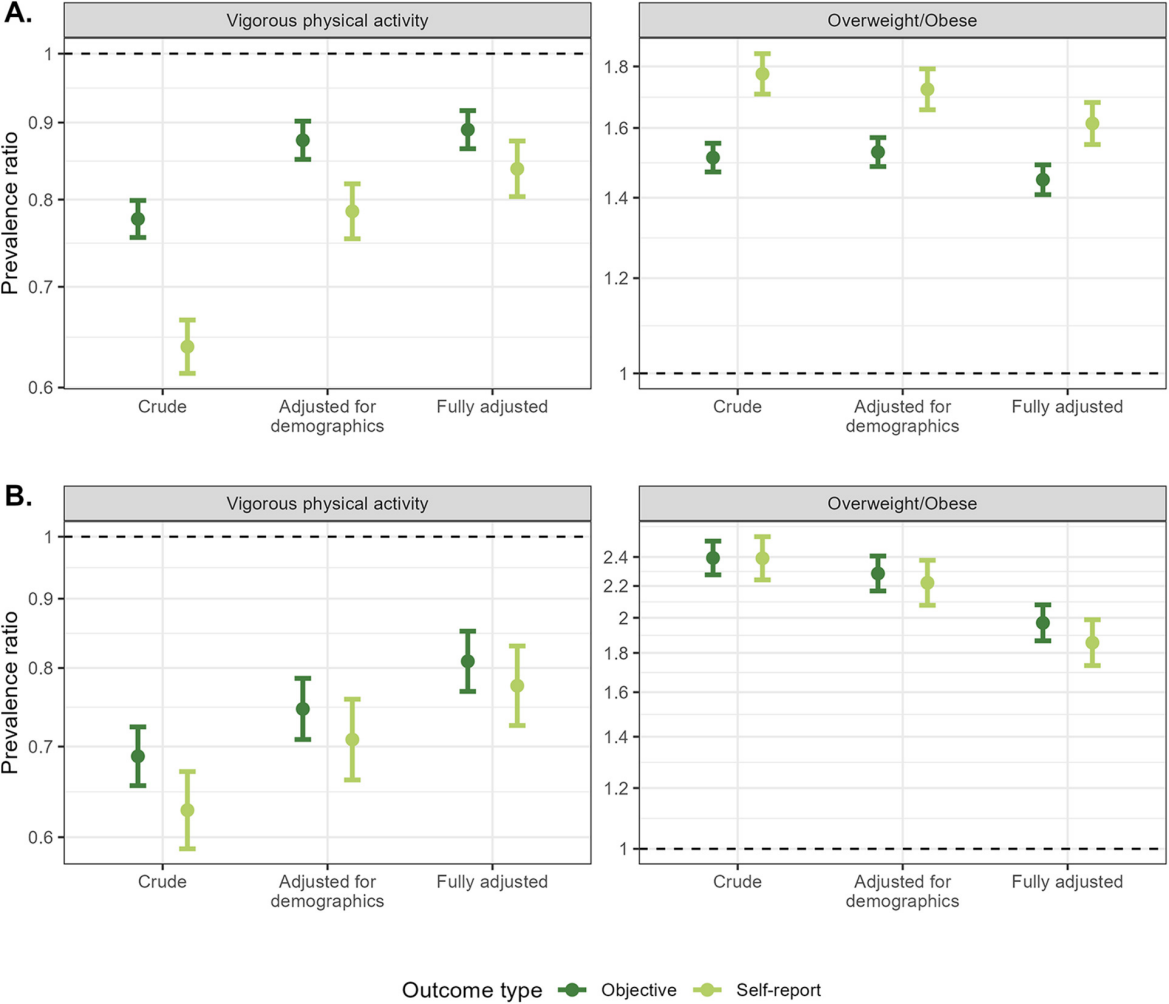
Comparisons of self-reported and objective high blood pressure **(A)** and diabetes **(B)** in models assessing the association between both a self-report (vigorous physical activity) and objective (overweight or obese BMI) exposure and high blood pressure and diabetes. Models adjusted for demographics controlled for age (spline), gender, and educational attainment. Fully adjusted models additionally controlled for state, rural/urban residence, marital status, literacy, self-reported smoking, and caste.

Vigorous physical activity was associated with lower prevalence of high blood pressure and diabetes; therefore, smaller prevalence ratios indicate stronger associations. Associations between vigorous physical activity and self-reported high blood pressure or diabetes were stronger using self-reported measures of high blood pressure and diabetes compared with objective measures (Figure 2). Differences were larger for crude and minimally adjusted estimates than for fully adjusted estimates. For example, the associations between vigorous physical activity and blood pressure were 77.30%, 82.04%, and 51.47% stronger when using self-reported rather than to objective measures of blood pressure for crude, minimally adjusted, and fully adjusted models. A similar pattern was observed for the association between overweight or obesity and high blood pressure but not for overweight or obesity and diabetes. This observed discrepancy was likely due to the fact that there was a large difference in the sensitivity for high blood pressure between those who were overweight or obese (0.593) compared with those with normal BMI (0.441), whereas the same magnitude of difference was not observed for diabetes (overweight or obese: 0.591, normal BMI: 0.553).

### 3.6 Impact when self-report measures were considered as confounders

**Figure 3:**
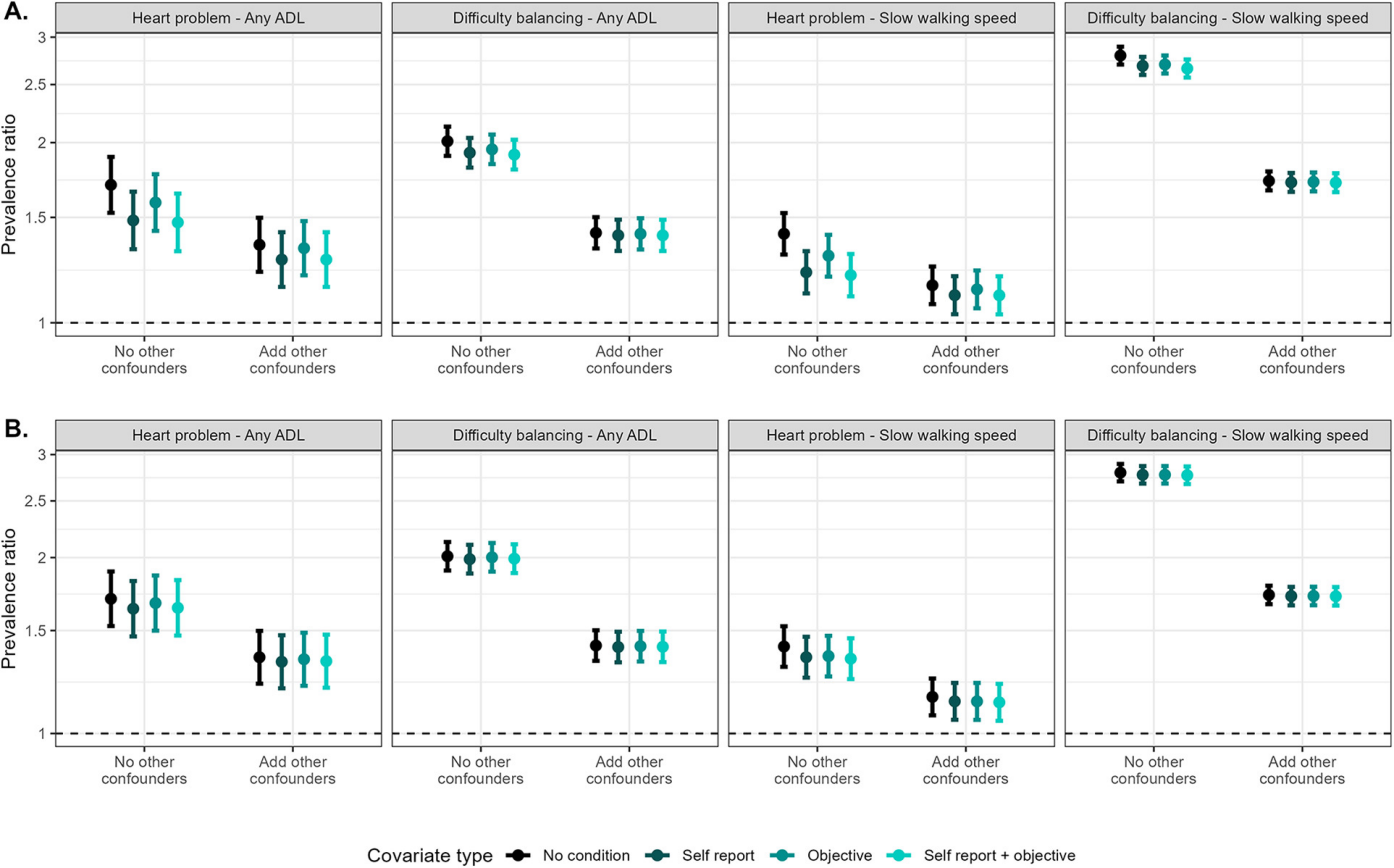
Comparisons of models including self-reported and objective high blood pressure **(A)** and diabetes **(B)** as hypothesized confounders and covariates in models assessing the associations between self-reported (any heart problem) and objective (difficulty balancing) exposures and self-reported [any activity of daily living (ADL) difficulty] and objective (slow walking speed) outcomes. Models that added other confounders as covariates additionally include age (spline), gender, educational attainment, state, rural/urban residence, marital status, literacy, self-reported smoking, caste, and BMI category.

Estimates were largely similar when comparing use of self-reported and objective measures of blood pressure and diabetes as confounders (Figure 3). The largest observed differences were for models examining heart problems as an exposure and using self-reported vs. objective blood pressure as a confounder. In these models, controlling for self-reported blood pressure attenuated estimates further toward the null compared with controlling for objective blood pressure. However, this attenuation was larger in models that did not adjust for other confounders compared with models that did adjust for other confounders. For example, when examining the association between self-reported heart problems and any ADL difficulty, estimates controlling for self-reported blood pressure or both self-reported and objective blood pressure were 43.25% and 46.47% weaker than the crude estimate, whereas the estimate controlling for objective blood pressure was 24.63% weaker. In models also controlling for other hypothesized confounders, the attenuation decreased to 26.37% and 26.66% for models controlling for self-reported or both self-reported and objective blood pressure and 10.80% when controlling for objective blood pressure.

### 3.7 Sensitivity analyses

Using the 2017 AHA guidelines for blood pressure led to higher prevalence estimates for objective blood pressure and therefore lower estimates of sensitivity and negative predictive value and higher specificity and positive predictive value for self-reported data. When using blood pressure as an exposure or outcome, differences between estimates using self-reported and objective data were larger than in primary analyses when considering blood pressure as either the exposure or outcome. Differences may be due to the fact that the broader AHA criteria include more mild cases, whereas self-report likely captures more severe cases. Differences were minimal when considering high blood pressure as a confounder.

20.22% of the unweighted sample reported use of blood pressure medications, comprising 24.16% of the group classified as having objective high blood pressure. 10.29% of the unweighted sample reported use of diabetes medications, comprising 16.84% of the group classified as having objective diabetes. Using objective measures of high blood pressure and diabetes that did not consider self-reported treatment status reduced the concordance between self-report and objective measures and increased the observed level of bias in example analyses. In comparison, results presented in primary analyses are more conservative.

## 4 Discussion

The use of self-reported vs. objective measures of cardiometabolic conditions can have a meaningful impact on results from epidemiologic studies in low- and middle-income contexts such as India. When considering high blood pressure or diabetes as either exposures or outcomes, using self-reported measures led to anti-conservative bias in effect estimates compared with objective measures across almost all analyses considered. We also observed one instance in which using self-reported rather than objective high blood pressure changed the statistical significance of findings, showing that observed biases can alter qualitative inferences from studies. The impact of using self-report instead of objective measures of cardiometabolic conditions as confounders was smaller, and attenuation of estimates compared with unadjusted results was larger using self-report than objective measures of cardiometabolic conditions.

The estimated sensitivity and specificity of self-reported high blood pressure and diabetes in this sample are on the lower end of the range of previously reported values (6–11), underscoring the importance of measurement error in this context. Results on differential performance of self-report are in line with prior studies showing that the sensitivity of self-report measures is lower for younger participants, men (6, 9, 21), and those with lower education (6, 8–10, 12, 15). Estimates of differential sensitivity and specificity or positive and negative predictive values can be used as inputs in quantitative bias analyses for misclassification in future studies. In addition to providing information on the expected direction of potential bias, algebraic adjustments based on these estimates could be used to quantify potential bias in subsequent analyses of associations or hypothesized causal effects.

Our results also agree with a prior study from Canada on the impact of using self-reported high blood pressure on estimated associations, which found that using self-reported rather than objective high blood pressure as an outcome in analyses overestimated effect estimates (21). The authors of this prior study argued that bias in the estimated association between high blood pressure and obesity may be because obese individuals were more likely to be aware of their blood pressure status. However, this explanation is unlikely to explain biases observed across all exposure/outcome pairs considered in our analysis. For example, in our study, while we replicated the prior finding on the association between BMI and self-reported vs. objective blood pressure, the same anti-conservative bias was also observed in the self-reported exposure considered (vigorous physical activity). However, self-reported vigorous physical activity is less likely to be related to disease awareness, and therefore the biasing mechanism is likely different.

Although different mechanisms across may underlie the observed bias across the exposure/outcome pairs considered, anti-conservative biases observed across analyses are likely due to higher sensitivity of self-report measures among the exposed compared with the unexposed. One alternative explanation is same-source bias, which describes bias due to correlation in measurement error (dependent measurement error) in the exposure and the outcome (29). This type of bias has been described across fields (30–32) and is common when the exposure and outcome are measured using the same source (i.e., self-report); those who self-report issues in one domain of health (cardiovascular disease) may also be more likely to report issues in another domain of health (i.e., ADL limitations). Same-source bias has also been hypothesized as an explanation for the observed bias in the association between self-reported hearing loss and self-reported outcomes in epidemiologic research (20). However, this hypothesized mechanism cannot explain observed anti-conservative bias in this study when the outcome of interest (when self-reported cardiometabolic conditions were exposures) or exposure of interest (when self-reported cardiometabolic conditions were outcomes) was measured objectively. In these scenarios, bias could be due to differential awareness, as described in the BMI example, or could be due to differential disease severity among those who are aware vs. unaware of their disease status. If those who self-reported having high blood pressure or diabetes are more likely have more severe disease, estimated associations with objective health outcomes may be stronger for self-reported measures.

Compared with the observed anti-conservative bias when considering self-reported cardiometabolic conditions as exposures or outcomes, when we considered self-reported vs. objective cardiometabolic conditions as confounders in analyses, observed biases were smaller. Differences in estimates were even more negligible when adding other hypothesized confounders to models to assess the impacts in more realistic modeling scenarios. Although the magnitude of differences was small, the attenuation of estimates was somewhat larger (indicating that the variable was a stronger confounder) when using self-report rather than objective measures of blood pressure and diabetes, particularly when considering a self-reported exposure (self-reported heart problems). This observed pattern may also be due to same-source bias: using a self-report measure as a confounder may control for the correlation in measurement error between the exposure and outcome (29). This result suggests that controlling for self-report measures to adjust for an individuals' latent tendency to respond positively to self-report survey measures may help reduce bias in studies subject to dependent measurement error. However, the magnitude of attenuation observed when controlling for self-reported cardiometabolic conditions was much smaller than the magnitude of anti-conservative bias hypothesized to be due to same-source error.

Some limitations should be considered. First, we conducted a complete case analysis, excluding those with missing data on any of the considered covariates of interest. However, the primary goal of this study was not to make generalizable conclusions about health conditions in India, but rather to compare the impact of using self-report vs. objective measures of cardiometabolic conditions. Although sample exclusions may have some impact if those excluded differ from those included on factors that affect the performance of self-reported cardiometabolic conditions, differences between those included and excluded from our sample were generally small. Given the magnitude of observed bias, overall conclusions are unlikely to be affected. Second, while the included objective measure of diabetes (based on HbA1c) was considered the gold standard in the current analysis, other measures (e.g., fasting plasma glucose) add additional information relevant to the characterization and diagnosis of diabetes but were not available in the current study. Despite evidence of discordance between measures of HbA1c and fasting plasma glucose or other diabetes assessments (33), prior studies showing very low disease awareness suggest that differences between self-report and objective measures would be expected to be considerably larger than differences between different objective measurement procedures (34, 35). Therefore, substantive conclusions of the current study and broader comparisons between self-reported diabetes status and any of these objective measures would be unlikely to be meaningfully different. Third, while we assessed findings across many example analyses looking at associations between self-report and objective exposures and outcomes that may be of interest in epidemiologic research, analyses cannot be exhaustive, and patterns of findings may not generalize to all potential future analyses. However, results were largely consistent across most analyses considered. If variables of interest in future analyses are present in the publicly available LASI dataset or other public data resources, investigators should seek to replicate comparisons examined in this paper using the variables of interest in their study to confirm the expected direction of bias.

In summary, this study contributes to the evidence on the performance of self-report measures of blood pressure and diabetes compared with objective measures. Low sensitivity of self-report measures due to lack of awareness is a major issue in low-income settings, and bias is likely to be differential by educational attainment and other SES-related factors. In this study, we found that substantial anti-conservative bias may exist when using self-reported cardiometabolic conditions as either exposures or as outcomes in epidemiologic research, regardless of whether other variables considered in analyses are self-reported or objectively measured. This has important consequences for existing and future research in low- and middle-income settings similar to India. Objective measures of cardiometabolic conditions should be used when possible, and use of methodologic innovations, including quantitative bias analysis (36), may help researchers understand the potential effects of biases on findings from studies using self-report measures.

## Data Availability

Publicly available datasets were analyzed in this study. This data can be found here: Gateway to Global Aging Research (https://g2aging.org/app/home).

## References

[1] Kelly BB Narula J Fuster V. Recognizing global burden of cardiovascular disease and related chronic diseases. Mt Sinai J Med. (2012) 79:632–40. 10.1002/msj.21345 23239202

[2] Lawes CM Hoorn SV Rodgers A. Global burden of blood-pressure-related disease, 2001. Lancet. (2008) 371:1513–8. 10.1016/S0140-6736(08)60655-8 18456100

[3] Cho NH Shaw JE Karuranga S Huang Y da Rocha Fernandes JD Ohlrogge AW IDF diabetes atlas: global estimates of diabetes prevalence for 2017 and projections for 2045. Diabetes Res Clin Pract. (2018) 138:271–81. 10.1016/j.diabres.2018.02.023 29496507

[4] Omran AR. The epidemiologic transition theory revisited thirty years later. World Health Stat Q. (1998) 53:99–119. 10.1111/j.1468-0009.2005.00398.x

[5] Chesnais J-C. Demographic transition patterns and their impact on the age structure. Popul Dev Rev. (1990) 16:327–36. 10.2307/1971593

[6] Najafi F Pasdar Y Shakiba E Hamzeh B Darbandi M Moradinazar M Validity of self-reported hypertension and factors related to discordance between self-reported and objectively measured hypertension: evidence from a cohort study in Iran. J Prev Med Public Health. (2019) 52:131–9. 10.3961/jpmph.18.257 30971080 PMC6459766

[7] Williams J Duncan D Cantor J Elbel B Ogedegbe G Ravenell J. A comparison of measured and self-reported blood pressure Status among low-income housing residents in New York city. J Health Dispar Res Pract. (2017) 9:153–65. Available online at: https://digitalscholarship.unlv.edu/jhdrp/vol9/iss4/9

[8] Ning M Zhang Q Yang M. Comparison of self-reported and biomedical data on hypertension and diabetes: findings from the China health and retirement longitudinal study (CHARLS). BMJ Open. (2016) 6:e009836. 10.1136/bmjopen-2015-009836 26729390 PMC4716227

[9] Chun H Kim I-H Min K-D. Accuracy of self-reported hypertension, diabetes, and hypercholesterolemia: analysis of a representative sample of Korean older adults. Osong Public Health Res Perspect. (2016) 7:108–15. 10.1016/j.phrp.2015.12.002 27169009 PMC4850372

[10] Goldman N Lin I-F Weinstein M Lin Y-H. Evaluating the quality of self-reports of hypertension and diabetes. J Clin Epidemiol. (2003) 56:148–54. 10.1016/S0895-4356(02)00580-2 12654409

[11] Burvill AJ Murray K Knuiman MW Hung J. Comparing self-reported and measured hypertension and hypercholesterolaemia at standard and more stringent diagnostic thresholds: the cross-sectional 2010–2015 Busselton healthy ageing study. Clinical Hypertension. (2022) 28:16. 10.1186/s40885-022-00199-1 35642010 PMC9158272

[12] Mulyanto J Kringos DS Kunst AE. The accuracy of self-report versus objective assessment for estimating socioeconomic inequalities in disease prevalence in Indonesia. Int J Public Health. (2019) 64:1233–41. 10.1007/s00038-019-01301-5 31531681 PMC6811380

[13] Delhey L Shoults C Johnson K Orloff M Faramawi MF Delongchamp R. The difference between hypertension determined by self-report versus examination in the adult population of the USA: continuous NHANES 1999–2016. J Public Health. (2021) 43:316–24. 10.1093/pubmed/fdz132 31781770

[14] Johnston DW Propper C Shields MA. Comparing subjective and objective measures of health: evidence from hypertension for the income/health gradient. J Health Econ. (2009) 28:540–52. 10.1016/j.jhealeco.2009.02.010 19406496

[15] Taylor A Dal Grande E Gill T Pickering S Grant J Adams R Comparing self-reported and measured high blood pressure and high cholesterol status using data from a large representative cohort study. Aust N Z J Public Health. (2010) 34:394–400. 10.1111/j.1753-6405.2010.00572.x 20649780

[16] Mosca I Bhuachalla BN Kenny RA. Explaining significant differences in subjective and objective measures of cardiovascular health: evidence for the socioeconomic gradient in a population-based study. BMC Cardiovasc Disord. (2013) 13:64. 10.1186/1471-2261-13-64 24119371 PMC3765886

[17] Bhatia M Dixit P Kumar M Dwivedi LK. Comparing socio-economic inequalities in self-reported and undiagnosed hypertension among adults 45 years and over in India: what explains these inequalities? Int J Equity Health. (2023) 22:26. 10.1186/s12939-023-01833-6 36732766 PMC9893593

[18] Rothman KJ Greenland S Lash TL. Modern Epidemiology. Philadelphia, PA:Wolters Kluwer Health/Lippincott Williams & Wilkins Philadelphia (2008).

[19] Tucker JM Welk GJ Beyler NK Kim Y. Associations between physical activity and metabolic syndrome: comparison between self-report and accelerometry. Am J Health Promot. (2016) 30:155–62. 10.4278/ajhp.121127-QUAN-576 25806568

[20] Choi JS Betz J Deal J Contrera KJ Genther DJ Chen DS A comparison of self-report and audiometric measures of hearing and their associations with functional outcomes in older adults. J Aging Health. (2016) 28:890–910. 10.1177/0898264315614006 26553723 PMC5937530

[21] Chen Y Rennie DC Lockinger LA Dosman JA. Association between obesity and high blood pressure: reporting bias related to gender and age. Int J Obes. (1998) 22:771–7. 10.1038/sj.ijo.0800658 9725637

[22] Onur I Velamuri M. The gap between self-reported and objective measures of disease status in India. PLoS One. (2018) 13:e0202786. 10.1371/journal.pone.0202786 30148894 PMC6110485

[23] Satheesh G Dhurjati R Balagopalan JP Mohanan PP Salam A. Comparison of Indian clinical practice guidelines for the management of hypertension with the world health organization, international society of hypertension, American, and European guidelines. Indian Heart J. (2024) 76:6–9. 10.1016/j.ihj.2023.12.009 38171390 PMC10943557

[24] Whelton PK Carey RM Aronow WS Casey DE Collins KJ Dennison Himmelfarb C 2017 ACC/AHA/AAPA/ABC/ACPM/AGS/APhA/ASH/ASPC/NMA/PCNA guideline for the prevention, detection, evaluation, and management of high blood pressure in adults: a report of the American College of Cardiology/American Heart Association task force on clinical practice guidelines. J Am Coll Cardiol. (2018) 71:e127–248. 10.1016/j.jacc.2017.11.006 29146535

[25] Hu P Edenfield M Potter A Kale V Risbud A Williams S Validation and modification of dried blood spot-based glycosylated hemoglobin assay for the longitudinal aging study in India. Am J Hum Biol. (2015) 27:579–81. 10.1002/ajhb.22664 25472916 PMC4454625

[26] American Diabetes Association. Classification and diagnosis of diabetes: standards of medical care in diabetes—2018. Diabetes Care. (2017) 41:S13–27. 10.2337/dc18-S002 29222373

[27] World Health Organization. A healthy lifestyle—WHO recommendations. (2010). Available online at: https://www.who.int/europe/news-room/fact-sheets/item/a-healthy-lifestyle—who-recommendations (Accessed December 20, 2023).

[28] Katz S. Assessing self-maintenance: activities of daily living, mobility, and instrumental activities of daily living. J Am Geriatr Soc. (1983) 31:721–7. 10.1111/j.1532-5415.1983.tb03391.x 6418786

[29] Ranker LR Petersen JM Fox MP. Awareness of and potential for dependent error in the observational epidemiologic literature: a review. Ann Epidemiol. (2019) 36:15–19.e2. 10.1016/j.annepidem.2019.06.001 31402082

[30] Chan D. “So why Ask Me? Are Self-Report Data Really That Bad?”, Statistical and Methodological Myths and Urban Legends. New York: Routledge (2008).

[31] Del Boca FK Noll JA. Truth or consequences: the validity of self-report data in health services research on addictions. Addiction. (2000) 95:347–60. 10.1046/j.1360-0443.95.11s3.5.x 11132362

[32] Podsakoff PM MacKenzie SB Podsakoff NP. Sources of method bias in social science research and recommendations on how to control it. Annu Rev Psychol. (2012) 63:539–69. 10.1146/annurev-psych-120710-100452 21838546

[33] Gujral UP Prabhakaran D Pradeepa R Kandula NR Kondal D Deepa M Isolated HbA1c identifies a different subgroup of individuals with type 2 diabetes compared to fasting or post-challenge glucose in Asian Indians: the CARRS and MASALA studies. Diabetes Res Clin Pract. (2019) 153:93–102. 10.1016/j.diabres.2019.05.026 31150721 PMC6635041

[34] Salas A Acosta D Ferri CP Guerra M Huang Y Jacob KS The prevalence, correlates, detection and control of diabetes among older people in low and middle income countries. A 10/66 dementia research group population-based survey. PLoS One. (2016) 11:e0149616. 10.1371/journal.pone.0149616 26913752 PMC4767439

[35] Manne-Goehler J Geldsetzer P Agoudavi K Andall-Brereton G Aryal KK Bicaba BW Health system performance for people with diabetes in 28 low- and middle-income countries: a cross-sectional study of nationally representative surveys. PLoS Med. (2019) 16:e1002751. 10.1371/journal.pmed.1002751 30822339 PMC6396901

[36] Lash TL Fox MP MacLehose RF Maldonado G McCandless LC Greenland S. Good practices for quantitative bias analysis. Int J Epidemiol. (2014) 43:1969–85. 10.1093/ije/dyu149 25080530

