# Supplementary material for: The impact of using self-report versus objective measures of cardiometabolic conditions in epidemiologic research: a case study from India using data from the longitudinal aging study in India
Source: Front Epidemiol. 2025 Jul 25;4:1372972. doi: 10.3389/fepid.2024.1372972 (PMC12331483; doi:10.3389/fepid.2024.1372972)
Supplement: Supplementary file 1 [file Datasheet1.pdf]

Supplementary Material

The impact of using self-report versus objective measures of cardiovascular disease in epidemiologic research: a case study from India using data from the Longitudinal Aging Study in India (LASI)

Emma Nichols*, Peifeng Hu, David E Bloom, Jinkook Lee, TV Sekher

*** Correspondence:** Emma Nichols: emmanich@usc.edu

1 Description of Dried Blood Spot Weights

Weights were constructed in two steps. In the first step, a Longitudinal Aging Study in India (LASI) design weight was created to account for unequal selection probabilities of households and therefore individuals within selected households. In the second step, post-stratification weights were created to correct for differential nonresponse and willingness to provide a blood sample. The second step was aimed to align the sample of LASI respondents with dried blood spot (DBS) data with the reference population in terms of key sociodemographic variables. Specifically, starting from the design weight, a raking algorithm was applied to match the weighted distribution of specific sociodemographic variables in the LASI DBS sample with their population counterparts. The reference population was the Indian adult population aged 45 years and older, and benchmark distributions were obtained from the 2011 Indian Census. The following raking factors were used (1) gender (male, female) × age (<50, 50–59, 60–69, 70+), (2) a rural indicator (rural, urban), and (3) gender (male, female) × education (no school, primary or less, middle, secondary or more). The raking procedure was performed separately for each state and then scaled up to the size of the Indian population aged 45 years and older. Thus, the weights make the LASI DBS sample representative (in terms of gender, age, education, and urbanicity) of each state when the analysis is restricted to a specific state and of the overall population when the analysis uses the entire sample.

2 Supplementary Figures and Tables

2.1 Supplementary figures

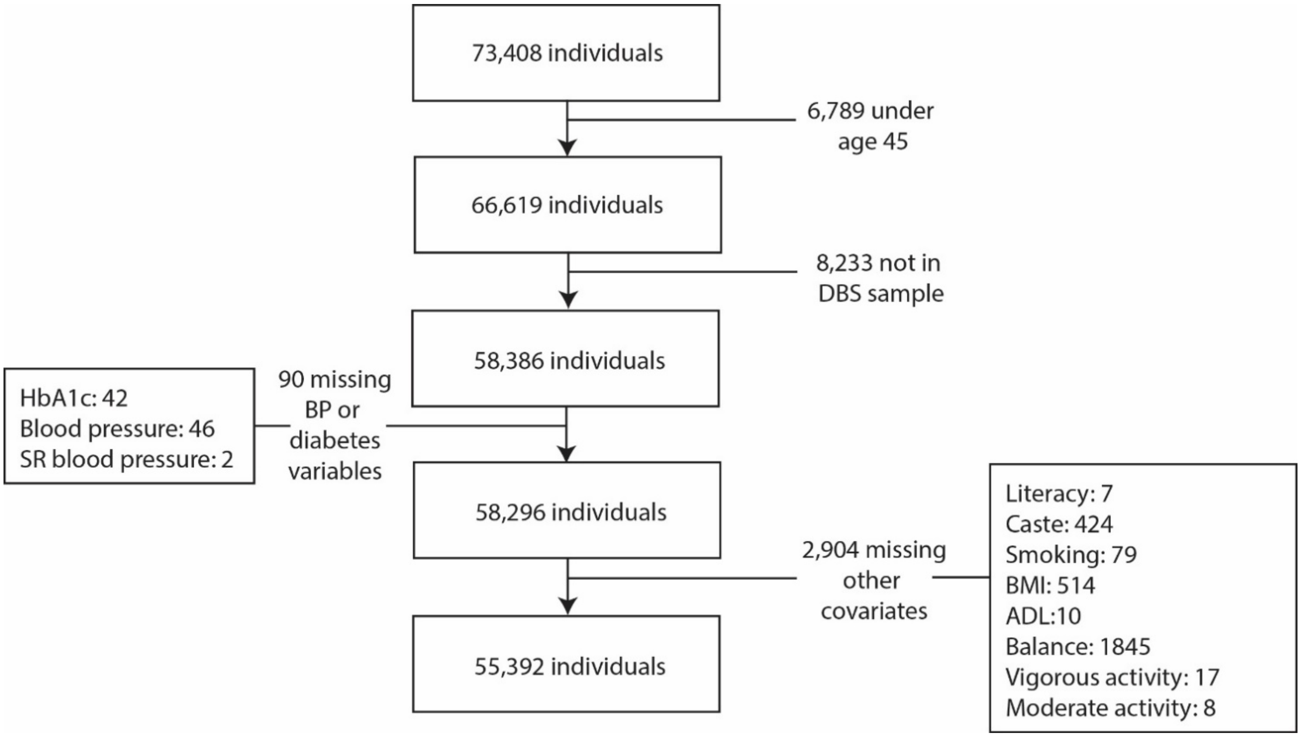

Supplementary Figure 1. Flowchart detailing sample exclusion criteria. DBS = dried blood spot. SR = self-report. BMI = Body-mass index. ADL = Activities of daily living.

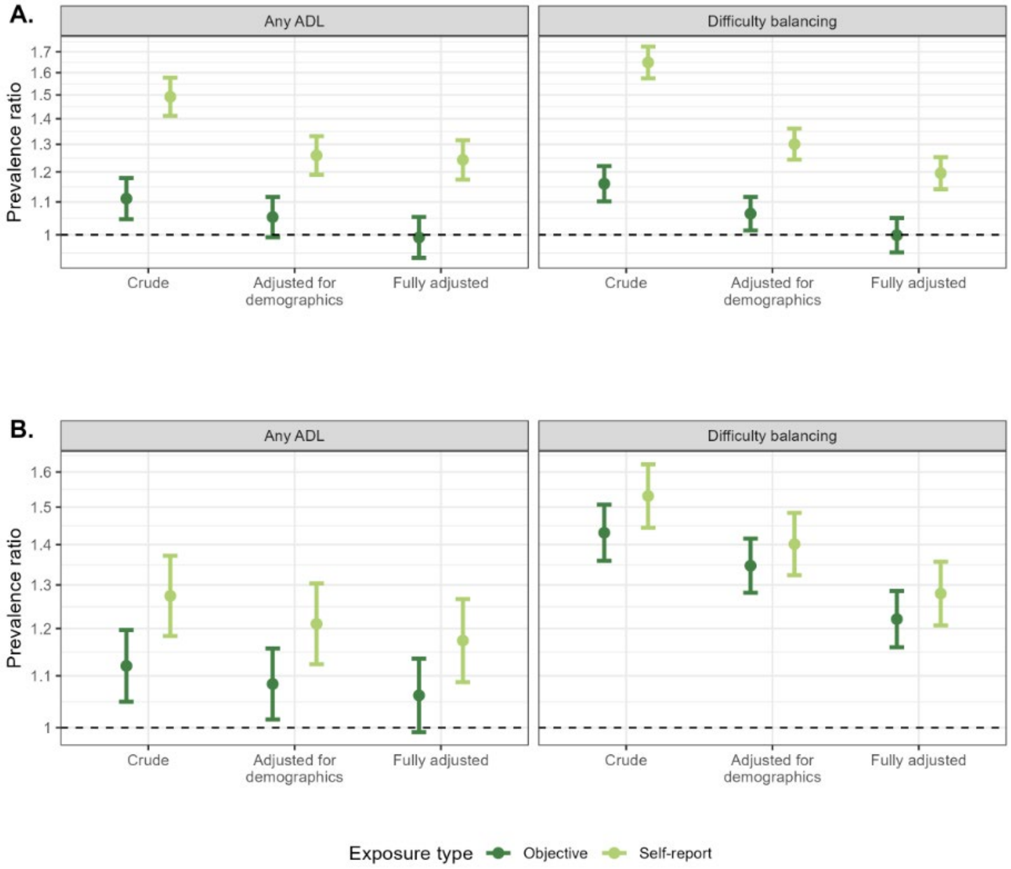

Supplementary Figure 2. Comparisons of self-reported and objective high blood pressure (using American Heart Association thresholds) [A] and diabetes [B] in models assessing the association between high blood pressure and diabetes with both a self-report (any Activity of Daily Living [ADL] difficulty) and objective (difficulty balancing) health outcome. Models adjusted for demographics controlled for age (spline), gender, and educational attainment. Fully adjusted models additionally controlled for state, rural/urban residence, marital status, literacy, self-reported smoking, caste, moderate and vigorous physical activity, and BMI category.

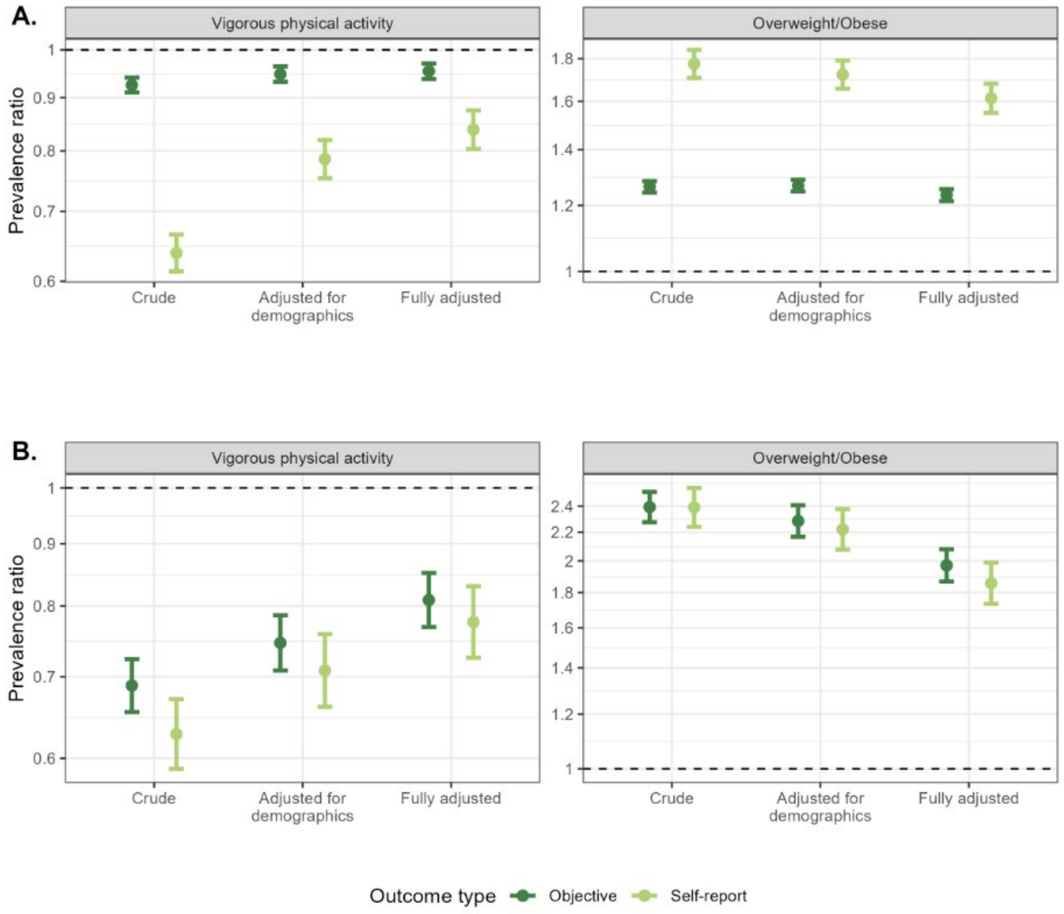

Supplementary Figure 3. Comparisons of self-reported and objective high blood pressure (using American Heart Association thresholds) [A] and diabetes [B] in models assessing the association between both a self-report (vigorous physical activity) and objective (overweight or obese BMI) exposure and high blood pressure and diabetes. Models adjusted for demographics controlled for age (spline), gender, and educational attainment. Fully adjusted models additionally controlled for state, rural/urban residence, marital status, literacy, self-reported smoking, and caste.

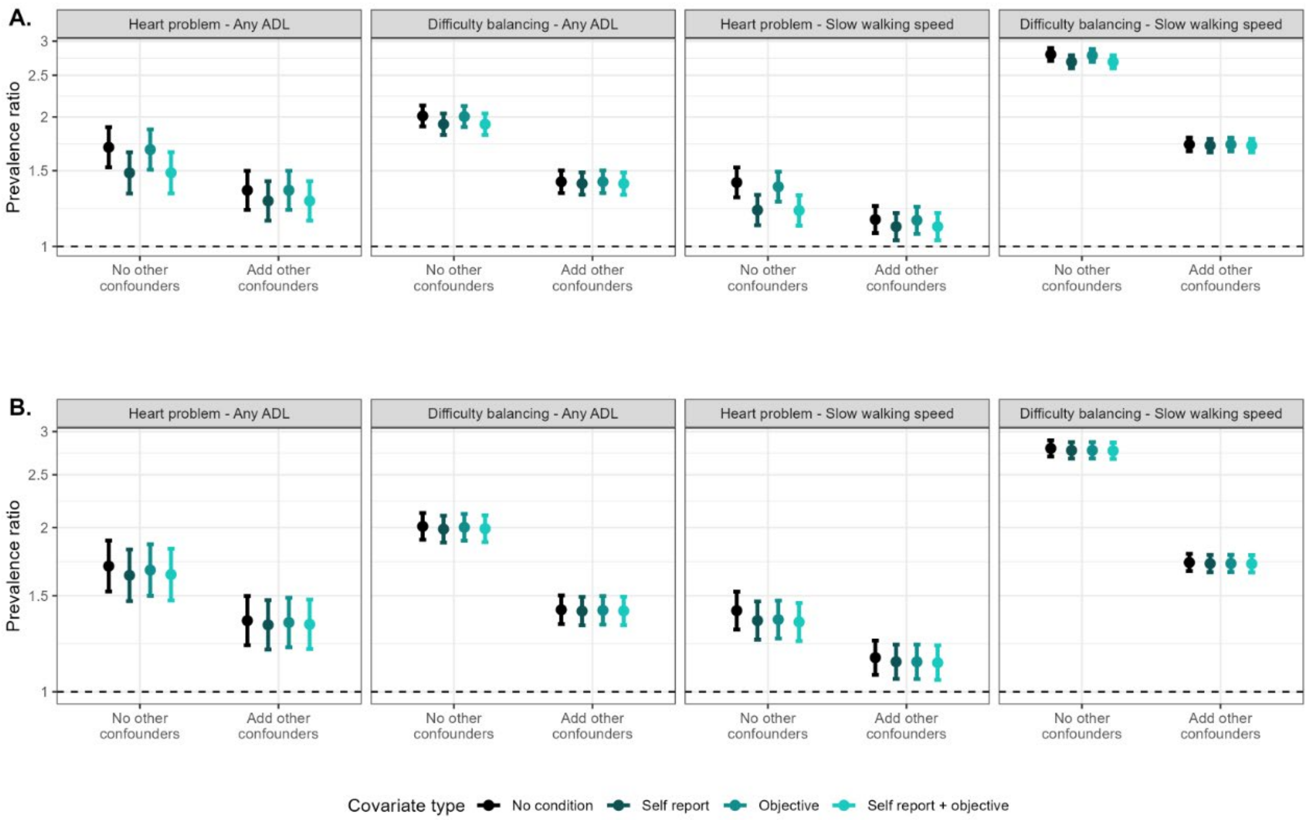

Figure 3. Comparisons of models including self-reported and objective high blood pressure (using American Heart Association thresholds) [A] and diabetes [B] as hypothesized confounders and covariates in models assessing the associations between self-reported (any heart problem) and objective (difficulty balancing) exposures and self-reported (any Activity of Daily Living [ADL] difficulty) and objective (slow walking speed) outcomes. Models that added other confounders as covariates additionally include age (spline), gender, educational attainment, state, rural/urban residence, marital status, literacy, self-reported smoking, caste, and BMI category.

2.2 Supplementary tables

Supplementary Table 1. Percentage difference for estimates of the association between blood pressure/diabetes and any activity of daily living (ADL) impairment/balancing difficulty comparing the use of self-reported and objective blood pressure and diabetes.

Outcome	Adjustment set	% difference for estimates of association comparing the use of self-reported and objective measures	
		Blood pressure	Diabetes
Any ADL	Crude	31.08	113.45
Any ADL	Adjusted for demographics	61.26	137.71
Any ADL	Fully adjusted	109.89	169.74
Difficulty balancing	Crude	18.86	18.81
Difficulty balancing	Adjusted for demographics	33.62	13.37
Difficulty balancing	Fully adjusted	43.22	23.54

* Models adjusted for demographics controlled for age (spline), gender, and educational attainment. Fully adjusted models additionally controlled for state, rural/urban residence, marital status, literacy, self-reported smoking, caste, moderate and vigorous physical activity and body-mass index category.

Supplementary Table 2. Percentage difference for estimates of the association between self-reported vigorous physical activity and overweight or obese body-mass index and blood pressure/diabetes comparing the use of self-reported and objective blood pressure and diabetes.

Exposure	Adjustment set	% difference for estimates of association comparing the use of self-reported and objective measures	
		Blood pressure	Diabetes
Vigorous physical activity	Crude	77.30	24.55
Vigorous physical activity	Adjusted for demographics	82.04	17.80
Vigorous physical activity	Fully adjusted	51.47	19.60
Overweight/Obese	Crude	38.78	-0.11
Overweight/Obese	Adjusted for demographics	28.32	-3.37
Overweight/Obese	Fully adjusted	29.04	-8.76

* Models adjusted for demographics controlled for age (spline), gender, and educational attainment. Fully adjusted models additionally controlled for state, rural/urban residence, marital status, literacy, self-reported smoking, and caste.

Supplementary Table 3. Percentage difference for estimates of associations between self-reported (any heart problem) and objective (difficulty balancing) exposures and self-reported (any activity of daily living [ADL] difficulty) and objective (slow walking speed) outcomes comparing crude models to models controlling for either self-reported or objective blood pressure and diabetes.

			% difference for estimates of association comparing the use of self-reported and objective measures					
			Blood pressure			Diabetes		
**Outcome**	**Exposure**	**Adjustment set**	Self-report	Objective	Self-report + Objective	Self-report	Objective	Self-report + Objective
Any ADL	Heart problem	No other confounders	-25.78	-12.80	-27.20	-7.37	-3.17	-6.71
Any ADL	Heart problem	Add other confounders	-18.95	-4.41	-18.96	-5.91	-2.61	-5.12
Any ADL	Difficulty balancing	No other confounders	-6.33	-4.45	-7.36	-1.64	-0.59	-1.37
Any ADL	Difficulty balancing	Add other confounders	-2.79	-1.18	-2.87	-1.66	-0.72	-1.39
Slow walking speed	Heart problem	No other confounders	-43.25	-24.63	-46.47	-12.26	-11.00	-13.98
Slow walking speed	Heart problem	Add other confounders	-26.37	-10.80	-26.66	-12.09	-12.23	-14.87
Slow walking speed	Difficulty balancing	No other confounders	-3.85	-3.33	-4.83	-0.84	-0.82	-0.99
Slow walking speed	Difficulty balancing	Add other confounders	-1.05	-0.69	-1.22	-0.83	-0.75	-0.97

*Models that added other confounders as covariates additionally include age (spline), gender, educational attainment, state, rural/urban residence, marital status, literacy, self-reported smoking, caste, and body-mass index category.

Note: given that all associations in this set of analyses were positive, negative percentage differences represent instances where the estimates were weaker after adjusting for a specific blood pressure or diabetes measure in comparison to the crude models.

Supplementary Table 4. The prevalence and performance of self-reported high blood pressure (using American Heart Association thresholds) and diabetes compared to objective measures of disease in the Longitudinal Aging Study in India (LASI) (N=55,392). 95% Confidence intervals are presented for estimates of prevalence. All reported statistics incorporate survey weights to account for the complex survey design.

	**Self-report prevalence**	**Objective prevalence**	**Sensitivity**	**Specificity**	**Positive predictive value**	**Negative predictive value**
**Blood pressure**						
Overall	25.7 (25.3 - 26.2)	67.0 (66.5 - 67.5)	0.346	0.923	0.901	0.411
Age category						
< 45	17.4 (15.5 - 19.4)	61.2 (58.6 - 63.8)	0.226	0.909	0.798	0.427

45-59	21.7 (21.1 - 22.3)	65.3 (64.5 - 66.0)	0.294	0.928	0.884	0.412
60-74	32.3 (31.5 - 33.1)	70.2 (69.4 - 71.0)	0.425	0.917	0.924	0.404
75+	35.1 (33.3 - 37.0)	70.5 (68.7 - 72.3)	0.463	0.917	0.930	0.416
Gender						
Men	21.1 (20.4 - 21.7)	67.1 (66.3 - 67.8)	0.286	0.944	0.913	0.394
Women	31.1 (30.4 - 31.7)	66.8 (66.1 - 67.5)	0.415	0.899	0.892	0.433
Rurality						
Urban	34.1 (33.2 - 34.9)	75.4 (74.5 - 76.2)	0.425	0.919	0.941	0.343
Rural	21.8 (21.3 - 22.3)	63.0 (62.4 - 63.6)	0.302	0.925	0.872	0.437
Education level						
No school	23.4 (22.8 - 24.1)	63.1 (62.4 - 63.9)	0.323	0.917	0.869	0.442
Less than secondary school	26.9 (25.9 - 27.8)	69.0 (68.0 - 70.0)	0.357	0.929	0.918	0.393
Secondary and higher secondary	28.3 (27.3 - 29.4)	72.1 (71.0 - 73.1)	0.370	0.940	0.941	0.366
Graduate school	33.0 (30.8 - 35.4)	76.1 (73.9 - 78.2)	0.406	0.909	0.934	0.325
**Diabetes**						
Overall	11.7 (11.4 - 12.0)	18.2 (17.8 - 18.6)	0.570	0.984	0.890	0.911
Age category						
< 45	6.5 (5.3 - 7.9)	14.1 (12.4 - 16.1)	0.418	0.993	0.910	0.912
45-59	10.3 (9.8 - 10.7)	16.9 (16.4 - 17.4)	0.541	0.986	0.890	0.914
60-74	14.9 (14.3 - 15.5)	21.1 (20.4 - 21.8)	0.627	0.979	0.886	0.907
75+	12.2 (11.0 - 13.5)	19.2 (17.7 - 20.8)	0.577	0.986	0.906	0.908
Gender						
Men	11.6 (11.1 - 12.1)	18.0 (17.4 - 18.6)	0.568	0.984	0.884	0.912
Women	11.8 (11.4 - 12.3)	18.5 (18.0 - 19.1)	0.573	0.985	0.898	0.910
Rurality						
Urban	19.3 (18.6 - 20.1)	28.2 (27.3 - 29.0)	0.637	0.981	0.928	0.873
Rural	8.1 (7.8 - 8.4)	13.6 (13.1 - 14.0)	0.505	0.986	0.848	0.927
Education level						
No school	8.0 (7.6 - 8.4)	13.6 (13.1 - 14.1)	0.495	0.986	0.846	0.925
Less than secondary school	13.7 (13.0 - 14.5)	20.7 (19.8 - 21.6)	0.598	0.983	0.901	0.904
Secondary and higher secondary	16.6 (15.8 - 17.5)	24.5 (23.6 - 25.5)	0.626	0.983	0.922	0.890
Graduate school	20.4 (18.5 - 22.4)	29.1 (26.9 - 31.3)	0.651	0.980	0.929	0.873
